# Global analysis reveals climatic controls on the oxygen isotope composition of cave drip water

**DOI:** 10.1038/s41467-019-11027-w

**Published:** 2019-07-05

**Authors:** Andy Baker, Andreas Hartmann, Wuhui Duan, Stuart Hankin, Laia Comas-Bru, Mark O. Cuthbert, Pauline C. Treble, Jay Banner, Dominique Genty, Lisa M. Baldini, Miguel Bartolomé, Ana Moreno, Carlos Pérez-Mejías, Martin Werner

**Affiliations:** 1 Connected Waters Initiative Research Centre, UNSW Sydney, Sydney, New South Wales 2052 Australia; 2 Chair of Hydrological Modeling and Water Resources, University of Freiburg, Freiburg, 79098 Germany; 3 Department of Civil Engineering, University of Bristol, Bristol, BS8 1TR UK; 4 Key Laboratory of Cenozoic Geology and Environment, Institute of Geology and Geophysics, Chinese Academy of Sciences, Beijing, 100029 China; 5 CAS Center for Excellence in Life and Paleoenvironment, 100044 Beijing, China; 6 ANSTO, Lucas Heights, New South Wales 2234 Australia; 7 School of Earth Sciences, University College Dublin, Belfield, Dublin, 4 Ireland; 8 School of Archaeology, Geography and Environmental Sciences, University of Reading, Whiteknights, Reading, RG6 6AH UK; 9 School of Earth and Ocean Sciences, Cardiff University, Cardiff, CF10 3AT UK; 10 Jackson School of Geosciences and Environmental Science Institute, The University of Texas at Austin, Austin, TX 78712 USA; 11 Environnements et Paléoclimats Océaniques et Continentaux, Université de Bordeaux, Pessac, 33615 France; 12 Department of Geography, Durham University, Durham, DH1 3LE UK; 13 Departamento de Procesos Geoambientales y Cambio Global, Instituto Pirenaico de Ecología-CSIC, Zaragoza, 50059 Spain; 14 Departamento de Geología, Museo Nacional de Ciencias Naturales (CSIC), Madrid, 28006 Spain; 15 Institute of Global Environmental Change, Xi’an Jiaotong University, 710049 Xi’an, China; 16 Alfred Wegener Institute, Helmholtz Centre for Polar and Marine Research, Bremerhaven, 27570 Germany

**Keywords:** Hydrology, Palaeoclimate

## Abstract

The oxygen isotope composition of speleothems is a widely used proxy for past climate change. Robust use of this proxy depends on understanding the relationship between precipitation and cave drip water δ^18^O. Here, we present the first global analysis, based on data from 163 drip sites, from 39 caves on five continents, showing that drip water δ^18^O is most similar to the amount-weighted precipitation δ^18^O where mean annual temperature (MAT) is < 10 °C. By contrast, for seasonal climates with MAT > 10 °C and < 16 °C, drip water δ^18^O records the recharge-weighted δ^18^O. This implies that the δ^18^O of speleothems (formed in near isotopic equilibrium) are most likely to directly reflect meteoric precipitation in cool climates only. In warmer and drier environments, speleothems will have a seasonal bias toward the precipitation δ^18^O of recharge periods and, in some cases, the extent of evaporative fractionation of stored karst water.

## Abstract (web summary)

δ^18^O of speleothems are a widely used paleoclimate proxy. Here, the authors conduct a global analysis of cave drip water δ^18^O compositions and find that drip waters from warmer climates have a seasonal bias toward precipitation δ^18^O of recharge periods, unlike in cooler climates where drip waters match well with recharge-weighted δ^18^O.

## Introduction

The oxygen isotope composition is by far the most widely reported climate proxy in cave deposits, or speleothems (e.g., stalagmites, stalactites and flowstones^1^). Multiple processes determine the oxygen isotope composition of speleothems (δ^18^O_speleo_), with the potential climate signal reflecting the source water (meteoric precipitation) δ^18^O (δ^18^O_precip_) and its relationship to local and regional climate. This signal is transferred to the cave through the vadose zone, where it may be mixed with existing waters and fractionated by evaporation. Finally, at the target (the speleothem), the δ^18^O_speleo_ signal can be further altered by non-equilibrium fractionation processes and temperature-dependent fractionation during calcite precipitation. See refs. ^1–3^ for in-depth reviews of these processes and climate signal transformation.

Within the speleothem research community, it is widely acknowledged that a cave monitoring approach is necessary to fully understand, and constrain quantitatively, the extent that the climate signal is preserved in δ^18^O_speleo_ (e.g., during transfer from the source to the target). The measurement of drip water hydrology^4^, drip water geochemistry^5^, cave environment^6^ and calcite growth and geochemistry^7^, as well as surface climate parameters, allows empirical relationships between the source and the target to be determined. With monitoring data, regression models between climate and speleothem proxy data can be developed^8^, proxy interpretations can be evaluated^9^, input data for forward or proxy system models can be generated^10–13^ and the extent that speleothem calcite precipitates in isotopic equilibrium with its associated drip water can be assessed^7,14,15^.

Recently, a new global database of speleothem carbon and oxygen isotope proxy records was compiled^16,17^. This archive includes 455 δ^18^O_speleo_ records, with over 324 covering intervals within the last 21 ka^16,17^. Some regions have δ^18^O_speleo_ records that span glacial–interglacial intervals (e.g. monsoon regions^18–20^), whereas other regions have records that are more complex (e.g. water-limited regions where δ^18^O_speleo_ exhibits high magnitude and frequency variability^21,22^). In water-limited environments, potential mechanisms by which δ^18^O_speleo_ can be modified during transit from the source, include evaporative fractionation of water δ^18^O in the soil; a shallow vadose zone or cave; selective recharge, whereby rainfall events with high amount or intensity have a distinct isotopic composition, typically low δ^18^O; non-equilibrium deposition during speleothem formation^23–27^. A fundamental research question is: what are the regional climate parameters where δ^18^O_speleo_ values most faithfully preserve the source signal (δ^18^O_precip_)? Identification of such climatic regions, and speleothem samples, will have the greatest utility; for example, for research methodologies, such as data assimilation^28^, which utilise proxy–climate model inter-comparison.

Interpretation of δ^18^O_speleo_ proxy records would benefit from the best possible understanding of the climatic conditions under which oxygen isotope composition of drip water (δ^18^O_dripwater_) is most directly related to δ^18^O_precip_. Here, we compile cave monitoring data with the objective of understanding the modern-day relationship between δ^18^O_precip_ and δ^18^O_dripwater_. We compile data sets where there are both cave δ^18^O_dripwater_ data (1-year or longer data sets) and δ^18^O_precip_ data (of equal duration, amount-weighted and collected close to the cave and similar altitude). The latter enables the amount-weighted precipitation oxygen isotope composition (δ^18^O_amountwprecip_) to be compared with δ^18^O_dripwater_. By using a karst hydrology model developed for European climates, monthly modelled recharge amount is used to obtain an annual recharge-weighted δ^18^O (δ^18^O_rechargewprecip_) at European sites. This permits the first analysis of δ^18^O_dripwater_, δ^18^O_rechargewprecip_, δ^18^O_amountwprecip_ and climate parameters. The analyses show that drip water δ^18^O is most similar to the amount-weighted precipitation δ^18^O, when mean annual temperature is < 10 °C. The implications for speleothem palaeoclimatology are that speleothems (if formed near isotopic equilibrium) are most likely to directly reflect meteoric precipitation δ^18^O only in cooler climates.

## Results

### Global water oxygen isotope distributions

**Fig. 1:**
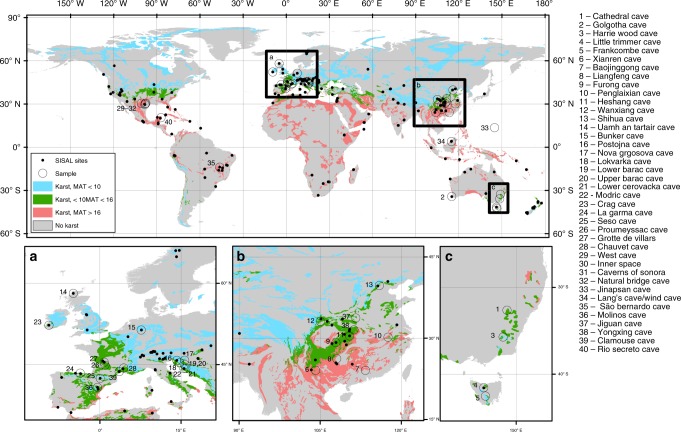
Global map of sample sites, karst regions, surface temperature and speleothem records. Location of the cave δ^18^O_dripwater_ samples (large circles). Global karst aquifer regions^41^ are shown as coloured areas, with those with mean annual temperature < 10 °C (blue); 10 °C < mean annual temperature < 16 °C (green) and mean annual temperature > 16 °C (red). Dots show the locations with speleothem (δ^18^O_speleo_) records in the SISAL (Speleothem Isotopes Synthesis and AnaLysis Working Group) database^16,17^. **a** Europe, **b** Chinese monsoon region and **c** SE Australia. More information on the sites is presented in Supplementary Data 1

We find a strong positive correlation between δ^18^O_dripwater_ and δ^18^O_amountwprecip_. δ^18^O_rechargewprecip_ provides a similarly strong correlation, but in this case with a slope and intercept indistinguishable from 1 and 0, respectively. Supplementary Data 1 presents the database of δ^18^O_dripwater_ and δ^18^O_amountwprecip_ compiled from the literature and unpublished data comprising 163 drip sites from 39 caves on five continents. The location of the caves in comparison with modern mean annual temperature (MAT) and the global database of δ^18^O_speleo_ records^17^ are shown in Fig. 1. Climate regimes represented in the compilation include temperate maritime and semiarid monsoon, Mediterranean, montane and tropical, therefore including a wide range of MAT and aridity, as expressed by the ratio of precipitation to potential evapotranspiration (P/PET).

**Fig. 2:**
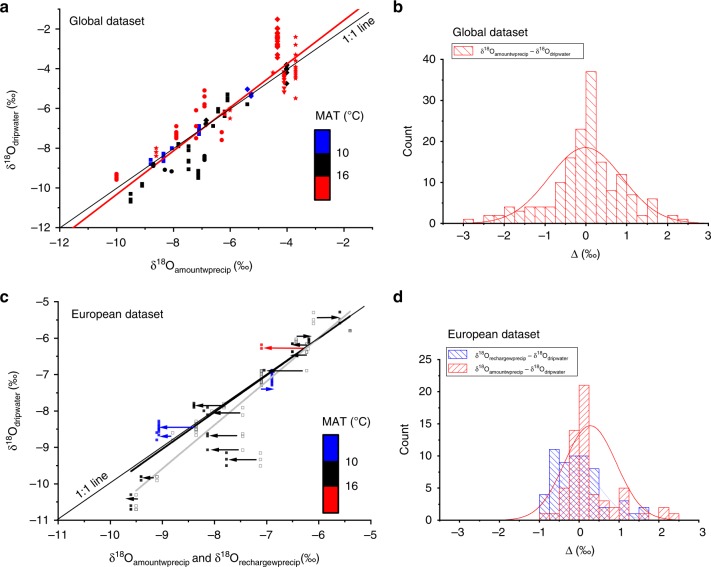
Global oxygen isotope relationships. **a** Global relationship between δ^18^O_dripwater_ and δ^18^O_amountwprecip_. The global data set regression line is shown in red: δ^18^O_dripwater_ = 0.64 (±0.25) + 1.10 ( ± 0.04) δ^18^O_amountwprecip_ (‰). Sites are coloured according to their mean annual temperature and symbols show their region: Europe (squares), China (circles), Australia (diamonds), United States (triangles) and other (stars). **b** Frequency histogram for the global data set for the difference between δ^18^O_amountwprecip_ and δ^18^O_dripwater_ (Δ_awp-dw_). **c** Relationship between the δ^18^O_rechargewprecip_, δ^18^O_amountwprecip_ and δ^18^O_dripwater_ for the European data set. The amount-weighted data are shown in open black symbols, and the regression line is shown in gray: *δ*^18^O_dripwater_ = 1.19 (±0.59) + 1.20 (±0.08) δ^18^O_amountwprecip_ (‰). The recharge-weighted data are shown by coloured symbols (as for (**a**)) and the regression line is shown in black: δ^18^O_dripwater_ = 0.06 (±0.50) + 1.01 (±0.06) δ^18^O_rechargewprecip_ (‰). The arrows show the direct effect of recharge weighting. **d** Frequency histogram for the European data set for the difference between δ^18^O_rechargewprecip_ and δ^18^O_dripwater_ (Δ_rwp-dw_) and δ^18^O_amountwprecip_ and δ^18^O_dripwater_ (Δ_awp-dw_) for the European data. Typical analytical uncertainties for individual δ^18^O analyses are ± 0.2‰^42^

Figure 2a, b presents the global relationship between δ^18^O_dripwater_ and δ^18^O_amountwprecip_. The correlation is positive and strong (Spearman’s rank *r*_s_ = 0.90, *p* < 0.00001), indicating that at a global scale, δ^18^O_dripwater_ closely relates to δ^18^O_amountwprecip_. The regression demonstrates that, at this scale, δ^18^O_dripwater_ is greater than δ^18^O_amountwprecip_ where the latter is more positive, typically sites where MAT > 16 °C. Conversely, δ^18^O_dripwater_ is less than δ^18^O_amountwprecip_ where the latter is more negative, typically at sites where MAT < 16 °C. Regional relationships between δ^18^O_dripwater_ and δ^18^O_amountwprecip_ for Europe, China and Australia are quantified in Supplementary Fig. 1. At a regional scale, the correlation is positive, very strong and highly significant for the European region and moderately strong for China.

For cave drip water monitoring sites in Europe, we utilise a karst hydrology model^29^ to determine the monthly recharge amount (see the ‘Methods’ section), and these monthly recharge values (see Supplementary Table 1) were then used to weight the δ^18^O_precip_ in that month. At the European scale, the relationship between the δ^18^O_dripwater_ and δ^18^O_amountwprecip_ is a strong positive correlation (Spearman’s rank *r*_s_ = 0.90, *p* < 0.00001), similar to that observed globally (Fig. 2c, d), although over a more restricted range of δ^18^O. With recharge weighting, the correlation between the δ^18^O_dripwater_ and δ^18^O_rechargewprecip_ remains positive and strong (Spearman’s rank *r*_s_ = 0.89, *p* < 0.00001). The intercept and gradient are indistinguishable from 0 to 1, respectively, indicating that after recharge weighting, at the European sites, δ^18^O_dripwater_ can be explained by δ^18^O_rechargewprecip_.

### Climate controls on selective recharge and partial evaporation

**Fig. 3:**
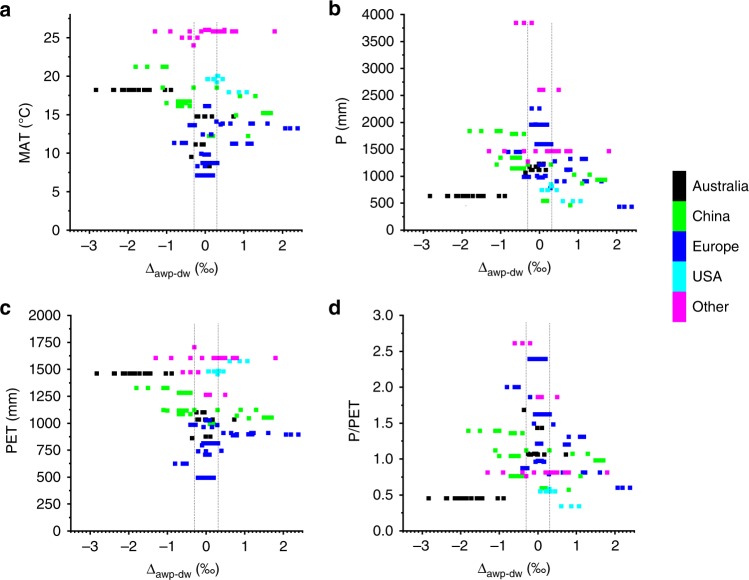
The global relationship between Δ_awp-dw_ and climate parameters. **a** Mean annual temperature (MAT), **b** total annual precipitation (P), **c** total annual potential evapotranspiration (PET), and **d** mean annual potential evapotranspiration relative to mean annual precipitation (P/PET). Colours represent different regions: Australia (black), China (green), Europe (blue), United States (cyan) and all other regions (magenta). Black vertical lines show the 0.3‰ criterion for determining the significant difference between δ^18^O_amountwprecip_ and δ^18^O_dripwater_

We provide empirical evidence from the global δ^18^O_dripwater_ data set that increasing temperature and decreasing rainfall both increase the absolute difference between δ^18^O_dripwater_ and δ^18^O_amountwprecip_. Figure 3 explores the global relationship between climate parameters and the difference between amount-weighted precipitation and drip water isotopic composition (Δ_awp-dw_ = δ^18^O_amountwprecip_ − δ^18^O_dripwater_). It can be observed that there is a narrowing in the range of Δ_awp-dw_ when MAT is relatively low (<10 °C), the total annual P is high (>1750 mm), the annual PET is low (<800 mm) or the total annual P/PET values are high (>1.5). Linear single and stepwise multiple regression analyses on the global data set showed that the strongest correlation (Spearman’s rank) of the absolute value of Δ_awp-dw_ was with the ratio of mean annual temperature (MAT) to the total annual P:

1 $$	|\Delta _{{\mathrm{awp - dw}}}| = 0.0106\left( { \pm 7.90439 \times 10^{ - 4}} \right) \\ 	\ \ + 0.00719\left( { \pm 8.75606 \times 10^{ - 4}} \right){\mathrm{MAT}}/P\left({{\,}^ \circ {\mathrm{mm}}^{ - 1}} \right)\\ 	\ \ \ \left( {r_{\mathrm{s}} = 0.51,\,p = 0.001072} \right)$$

To further explore the relationship between Δ_awp-dw_ and these climate parameters, we define a threshold for |Δ_awp-dw_| of more than 0.3‰ as a criterion for determining the significant difference between δ^18^O_amountwprecip_ and δ^18^O_dripwater_. This is chosen taking into consideration potential uncertainties in δ^18^O determinations of water and speleothem calcite (analytical uncertainties of 0.06–0.2‰, depending on measurement technique). Considering the climate parameter MAT, 91% of all drip waters with a MAT < 10 °C (*n* = 34) have a |Δ_awp-dw_| of <0.3‰. Considering the P, then for a P threshold of 1750 mm, 61% of all drip waters (*n* = 31) have a |Δ_awp-dw_| of < 0.3‰. These empirical observations agree with theoretical understanding that in warmer, water-limited climates, δ^18^O_dripwater_ may be affected by evaporative fractionation of the water in the soil or shallow karst^22,30^, or by selective recharge, with an isotopic composition dominated by those rainfall events or seasons that generate recharge^26,27^. However, we note that a combination of post-infiltration evaporative fractionation and isotopically depleted recharge could lead to observations of |Δ_awp-dw_| < 0.3‰ for some sites with warm and dry climates.

## Discussion

Our recharge modelling demonstrates the importance of selective recharge, and suggests that for a MAT < 16 °C, δ^18^O_dripwater_ is best interpreted as δ^18^O_rechargewprecip_. The 1:1 linearity of the relationship between δ^18^O_rechargewprecip_ and δ^18^O_dripwater_ for European sites confirms the importance of selective recharge for this climate range (seasonal climates with a MAT ranging from 7.1 to 16.1 °C, Supplementary Data 1). Selective recharge is minimised at MAT < 10 °C. At these temperatures, the opportunity for soil and shallow karst evaporation is decreased, and karst water stores are more likely to be maintained, allowing mixing of recharge waters that buffer the isotopic impact of any individual recharge event. At a MAT < 10 °C, speleothems that have been deposited close to equilibrium would have the potential of recording past variations of δ^18^O_amountwprecip_, plus a temperature signal from the fractionation during calcite precipitation.

Latitudes poleward of ~35° and high-altitude sites, where MAT < 10 °C (Fig. 1), would be most likely to contain a δ^18^O_speleo_ record of amount-weighted precipitation (northern Europe, high-altitude and northern regions of the Asian monsoon, northern North America and New Zealand). In contrast, δ^18^O_speleo_ records in regions of higher MAT are more likely to have |Δ_awp-dw_| > 0.3 ‰ and would be sensitive to moisture balance changes, due to limited mixing with stored water, selective recharge and/or increased chance of evaporative fractionation of δ^18^O in the vadose zone. δ^18^O_dripwater_, and the associated δ^18^O_speleo_, can be more positive than amount-weighted precipitation (evaporative fractionation dominates), or either greater or less than amount-weighted precipitation (selective recharge dominates). Regions where this compound signal is most likely are predominantly in latitudes equatorward of ~35° (most of Africa, India, southern Asia, southern Europe, North America and Australia; Fig. 1). Modelling of δ^18^O_rechargewprecip_ suggests that for seasonal climates with a MAT between 10 and 16 °C (the higher value being the upper bound of the European data set), selective recharge dominates these processes. At this range of MAT (and, we anticipate, at higher MAT), δ^18^O_speleo_ may be a proxy for δ^18^O_rechargewprecip_ and provides records of paleo-recharge. In addition, when considering δ^18^O_speleo_, any relationship between δ^18^O_dripwater_ and climate could be additionally overprinted by non-equilibrium deposition.

Our meta-analysis reveals that the oxygen isotope composition of drip water is primarily determined by the oxygen isotope composition of the recharge water δ^18^O. At a global scale, we show that the extent to which δ^18^O_dripwater_ is representative of δ^18^O_amountwprecip_ is primarily related to the mean annual temperature and annual precipitation, which determines the extent to which δ^18^O is further altered by soil and karst processes. To confidently interpret the δ^18^O_dripwater_ as a specific climate parameter, the relationship between recharge δ^18^O and climate needs to be understood for specific sites. For sites and regions, characterised by lower temperatures (MAT < 10 °C), where Δ_awp-dw_ is likely to be closest to zero, we show that the oxygen isotope composition of drip water is most directly related to the isotopic composition of local rainfall. These regions could produce δ^18^O_speleo_ proxies (if the speleothems are deposited close to equilibrium), where δ^18^O_speleo_ could be used to provide a signal of past δ^18^O_amountwprecip_ and cave air temperature (due to the temperature-dependent fractionation during calcite formation), useful for proxy–model assimilations. In these cooler climates, where water in karst stores and fractures is more likely to be well mixed, one would also expect greater agreement in δ^18^O_dripwater_ between drip sites within a cave. In regions with higher temperatures (MAT > 16 °C), δ^18^O_speleo_ is less likely to represent δ^18^O_rechargewprecip_, and instead can contain a compound signal that reflects selective recharge and evaporative fractionation. Such records are of palaeoclimatic value, and may yield a proxy for δ^18^O_rechargewprecip_, but are more likely to show greater heterogeneity between coeval records and therefore require a drip-specific interpretation.

Important Quaternary δ^18^O_speleo_ records have been produced from around the world, and in the context of this analysis of modern conditions, we can make several conclusions. Firstly, many palaeoclimate studies interpret the relative changes in δ^18^O_speleo_ over time, and in many cases, monitoring data are not available to guide the interpretation. The climatic controls made here can be used to help guide the interpretation of those records. This is particularly relevant over periods of significant climate change (e.g. glacial–interglacial transitions) and where the climate control on the difference between δ^18^O_amountwprecip_ and δ^18^O_dripwater_ may change over time. A map of the cave sites at modelled last glacial maximum (LGM) surface temperatures is provided in Supplementary Fig. 2, and suggests that a change in the temperature control on the δ^18^O_amountwprecip_–δ^18^O_dripwater_ relationship is mostly observed in mid-latitudes, and most ubiquitously in the LGM in southern Europe. Secondly, in the Chinese monsoon region, the cooler northern sites are most likely to have δ^18^O_dripwater_ similar to δ^18^O_amountwprecip_, as reported previously^30^. However, given that monsoon rainfall requires a land–ocean temperature gradient, there is a trade-off between caves at cooler locations that have δ^18^O_dripwater_ closest to δ^18^O_amountwprecip_, and those in regions with the strongest monsoon signal. The latter are more likely to experience evaporative fractionation and selective recharge, and therefore less likely to be similar to δ^18^O_amountwprecip_ (but may reflect δ^18^O_rechargewprecip_). This trade-off would apply to all monsoon regions. At the modern monitoring sites in the Chinese region, where MAT > 10 °C and annual P < 2000 mm (Fig. 3), δ^18^O_amountwprecip_ does not correlate with MAT or the total annual P, but δ^18^O_dripwater_ does positively correlate with both (Supplementary Fig. 3). This appears to be due to the combined overprinting of increasing selective recharge and evaporative fractionation over this range of MAT and offers new insights into the interpretation of δ^18^O_speleo_ in the region. Thirdly, even in regions of exceptionally high rainfall, such as Mulu (Malaysian Borneo) and parts of India, δ^18^O_dripwater_ can be higher than the δ^18^O_recharegewprecip_^31^, probably due to the continuous high temperatures, leading to the partial evaporation of vadose water. Analysis of speleothems at caves at higher elevations should help mitigate this effect. Finally, δ^18^O_speleo_ records from regions with high aridity and temperatures should not be expected to preserve a record of δ^18^O_precip_. Our meta-analysis confirms the modern monitoring observations^25^, which indicate that δ^18^O_speleo_ in these regions would be an archive of alternating palaeo-aridity and palaeo-recharge and supports the interpretation of δ^18^O_speleo_ as a palaeo-recharge and palaeo-aridity proxy for the last glacial maximum in arid southern Australia^22^.

## Methods

### Data compilation

δ^18^O_dripwater_ data were compiled from a literature search and unpublished data. To minimise uncertainties that could be introduced into our analysis, we have chosen to only include sites where both of the following two criteria were met. Firstly, δ^18^O_precip_ was collected at or close to the sites (<20 km) and at a similar altitude, monthly integrated samples for at least 1 year, with an amount-weighted annual mean (δ^18^O_amountwprecip_) value reported. Secondly, δ^18^O_dripwater_ was collected over the hydrological year, for at least 1 year, with at least bimonthly sampling frequency. Monitoring results had to have at least 1 year of both δ^18^O_dripwater_ and δ^18^O_precip_ data, with overlapping time periods. We therefore have not included sites where δ^18^O_precip_ is a derived parameter, e.g. from isotope-enabled GCM output or based on empirical relationship with distant Global Network of Isotopes in Precipitation (GNIP) stations. Average drip water age is unknown for all sites, and it is possible that for some locations, the δ^18^O_dripwater_ integrates δ^18^O_precip_ prior to the monitoring period.

For each site, the local MAT and the total annual P were taken from the publications, and PET was taken from the WorldClim Global Climate Database^32,33^. For one study^29^, the total annual precipitation was not provided, and output from the gridded data set was used instead. The P/PET was calculated from the local P and gridded PET.

### Climate comparison

δ^18^O_dripwater_ and δ^18^O_amountwprecip_ data were compared with the following climate characteristics: mean annual temperature (MAT), total annual precipitation (P), potential evapotranspiration (PET) and the precipitation over PET ratio or  aridity index (P/PET). PET and the P/PET were taken from the global aridity and PET database^32,33^, where PET is modelled at ~1-km resolution, using data from the WorldClim Global Climate Database using mean monthly extraterrestrial radiation, and mean monthly temperature and range (using the equation of ref. ^34^). Sites are classified as humid where P/PET > 0.65; semi-arid and dry sub-humid at 0.2 ≤ P/PET ≤ 0.65; arid and hyper-arid at P/PET < 0.2. The difference between the δ^18^O_amountwprecip_ and δ^18^O_dripwater_ was determined for each drip site (Δ_awp-dw_).

As cross-checks on the gridded database, we compared for all caves local P and gridded P (Eq. 2) and local T and gridded T (Eq. 3), and for the Australian caves, we compared gridded PET with the mean PET (1960–1990 AD) calculated from the Australian Water Availability Project (AWAP) database^35,36^:

2 $${\mathrm{Gridded}}\,{\mathrm{P}} = 1.04\,{\mathrm{P}}\left( {r = 0.98} \right)$$

3 $${\mathrm{Gridded}}\,{\mathrm{T}} = 1.00\,{\mathrm{T}}\left( {r = 0.96} \right)$$

For the Australian sites, the gridded PET calculated by the two products agreed within 7% for all sites, except Golgotha Cave, where the AWAP PET was 30% higher than that calculated by WorldClim. The difference in PET at this site did not change the P/PET classification (using WorldClim: 1.06; using AWAP 0.82), and the WorldClim data are used for consistency.

Statistical analyses were undertaken using Microcal Origin. Correlations are Spearman’s rank-correlation coefficients (*r*_s_). Probability values (*p*), are conservatively determined using the lowest degrees of freedom (df), based on the number of cave sites (global: *n* = 39; Europe: *n* = 16; China: *n* = 10; Australia: *n* = 5), rather than the number of unique drip waters. Regression equation slope and intercept uncertainties are the standard error.

### Karst hydrological model

To estimate recharge, we use a large-scale karst groundwater recharge model that was previously developed for European and Mediterranean climates^29,37,38^. The model simulates karstic groundwater recharge at a 0.25° × 0.25° resolution at a daily resolution for a 10-year period from 2002 to 2012, which we consider long enough to provide representative average values of monthly recharge. All relevant karstic and non-karstic processes, such as infiltration of rainfall and snowmelt, evapotranspiration, downward percolation from the upper soil layer to a lower soil/epikarst layer and vertical percolation from the epikarst layer towards the groundwater, are considered within its structure. The epikarst, which is a typical vadose-zone feature of karst systems, allows the dynamic separation of focused and diffuse groundwater recharge^38,39^. For the weighting of recharge, output from the epikarst is used: the epikarst in the model is a series of *N* = 15 linear storages with variable capacities (*V*_*soil,i*_ [mm] and *V*_*epi,i*_ [mm]) and with variable storage constants (*K*_*e*__*pi,i*_[d]), which are distributed by a Pareto function, with a shape parameter *a* [−]. Parameter estimation provided ranges of values for *V*_*soil,i*_ *V*_*epi,i*_*, K*_*epi*,*i*_ and *a* for the humid, mountain, Mediterranean and desert karst landscapes^29^. Here, we use the average recharge volumes (over all simulations obtained with the parameter sets within these confined ranges), and weight the δ^18^O_precip_ in each month by the fraction of the total annual epikarst recharge that occurred in that month.

## Supplementary information

Supplementary Information

Peer Review File

Description of Additional Supplementary Files

Supplementary Data 1

## Data Availability

All water isotope data presented in the figures are contained in the Supplementary Data and Table. The SISAL (Speleothem Isotopes Synthesis and AnaLysis Working Group) database version 1b, that supports Fig. 1 and Supplementary Fig. 3, is archived at the University of Reading. 10.17864/1947.189. The ECHAM5-wiso climate model data that support Supplementary Fig. 2 are archived at PANGAEA^40^.

## Code availability

The karst hydrology model code is deposited at https://github.com/KarstHub/VarKarst-R-2015.
