# Supplementary material for: Global analysis reveals climatic controls on the oxygen isotope composition of cave drip water
Source: Nat Commun. 2019 Jul 5;10:2984. doi: 10.1038/s41467-019-11027-w (PMC6611902; doi:10.1038/s41467-019-11027-w)
Supplement: Supplementary file 1 — Supplementary Information [file 41467_2019_11027_MOESM1_ESM.pdf]

Supplemental Information

Global analysis reveals climatic controls on the oxygen isotope composition of cave drip water

Baker A. et al.

The Supplementary information comprises one Supplementary Table, three Supplementary Figures and Supplementary References.

Supplementary Table 1.

Month	Uamh an Tartair, UK		Crag Cave, Ireland		La Garma Cave, Spain		Grotte de Villars, France		Chauvet Cave, France		Bunker Cave, Germany		Postojna Cave, Slovenia		Nova Grgosova Cave, Croatia	
	mean	sd	mean	sd	mean	sd	mean	sd	mean	sd	mean	sd	mean	sd	mean	sd
Jan	99.8	1.6	109.3	1.5	71.9	0.7	68.2	1.0	52.2	3.8	89.4	1.1	74.2	5.2	49.6	3.1
Feb	66.7	3.7	81.4	3.3	62.7	1.2	41.3	2.9	37.9	1.8	58.5	2.4	49.0	1.3	33.2	1.2
Mar	59.3	2.0	60.3	1.8	56.9	1.4	38.9	1.5	18.8	3.9	52.3	1.6	46.4	1.2	40.9	1.6
Apr	39.3	2.7	31.4	4.3	42.9	2.6	34.2	0.8	19.5	1.3	25.1	4.6	44.0	1.7	47.8	1.5
May	28.6	1.1	26.0	1.2	35.2	2.1	22.5	3.8	21.2	2.6	22.2	1.3	24.0	3.6	28.0	2.3
Jun	17.4	2.9	15.8	4.1	31.7	1.9	18.2	2.0	17.2	4.5	13.8	2.5	18.6	2.3	37.8	2.0
Jul	21.3	1.5	13.3	1.7	16.6	3.0	5.6	2.4	2.7	1.6	23.7	2.1	11.5	2.9	35.1	3.4
Aug	42.1	4.6	29.7	2.6	7.9	2.1	4.4	1.2	1.8	0.8	40.9	4.5	14.8	2.4	32.8	3.0
Sep	68.1	4.0	49.1	3.3	4.6	1.2	6.2	1.9	3.6	1.8	35.8	3.2	43.2	6.9	53.8	4.2
Oct	104.8	4.0	82.3	7.5	11.3	0.6	10.8	2.6	13.9	5.8	46.0	2.5	55.0	3.6	74.9	1.6
Nov	100.9	1.1	109.4	1.7	39.4	2.7	45.3	6.4	74.1	11.2	71.4	3.4	90.4	4.4	70.2	1.3
Dec	87.5	2.4	111.9	1.9	74.7	3.3	72.0	1.8	68.7	4.5	77.0	0.7	126.8	3.0	82.0	2.1
YEAR	736		1051		455		774		331		556		1065		586	
	Lokvarka Cave, Croatia		Lower Cerovačka Cave, Croatia		Upper/Lower Barać Cave, Croatia		Modrič Cave, Croatia		Proumeyssac Cave, France		Clamouse Cave, France		Molinos Cave, Spain		Seso Cave, Spain	
	mean	sd	mean	sd	mean	sd	mean	sd	mean	sd	mean	sd	mean	sd	mean	sd
Jan	72.9	3.2	82.1	4.5	95.0	4.7	103.4	6.9	57.3	1.0	52.6	1.9	20.6	2.0	22.8	1.4
Feb	45.1	2.3	55.7	2.2	53.9	1.7	62.0	3.0	32.1	3.7	42.5	2.7	20.4	1.8	17.3	1.6
Mar	43.9	1.1	52.3	1.2	48.4	0.9	54.2	2.3	24.6	1.6	23.4	3.7	22.0	2.1	13.2	1.8
Apr	53.3	0.4	67.0	1.1	51.4	1.5	48.6	1.3	30.2	1.7	22.1	3.5	38.0	3.7	15.2	0.8
May	37.0	3.1	58.5	1.9	33.0	3.7	35.1	3.3	20.4	3.7	26.3	4.8	49.2	4.4	20.9	1.2
Jun	29.0	2.9	63.7	2.3	34.0	2.1	28.1	2.9	11.5	2.3	13.6	4.2	14.3	4.0	10.3	2.8
Jul	14.6	4.3	36.7	5.1	12.4	3.7	15.3	3.7	3.3	1.8	4.2	1.9	6.1	2.4	5.1	2.0
Aug	18.9	2.1	38.7	2.0	13.7	2.9	9.5	2.6	2.8	1.1	3.1	1.4	6.1	2.3	2.8	1.3
Sep	47.0	3.0	54.2	1.7	30.0	2.7	21.9	1.6	2.6	1.1	27.2	7.2	16.2	3.9	2.0	1.0
Oct	73.5	2.9	73.4	3.4	45.6	4.5	38.2	2.4	8.5	2.3	53.6	10.1	26.0	4.2	3.3	1.5
Nov	86.5	2.3	90.9	2.8	76.1	6.2	64.1	3.2	23.7	5.0	83.3	9.1	32.1	3.3	10.8	3.2
Dec	105.6	3.5	105.2	2.7	122.9	2.9	119.7	6.9	51.3	3.2	60.3	5.8	25.9	1.7	18.2	2.4
YEAR	627		778		616		600		268		412		277		391	

Modelled monthly recharge (mm per month).

Supplementary Figures

Supplementary Figure 1.

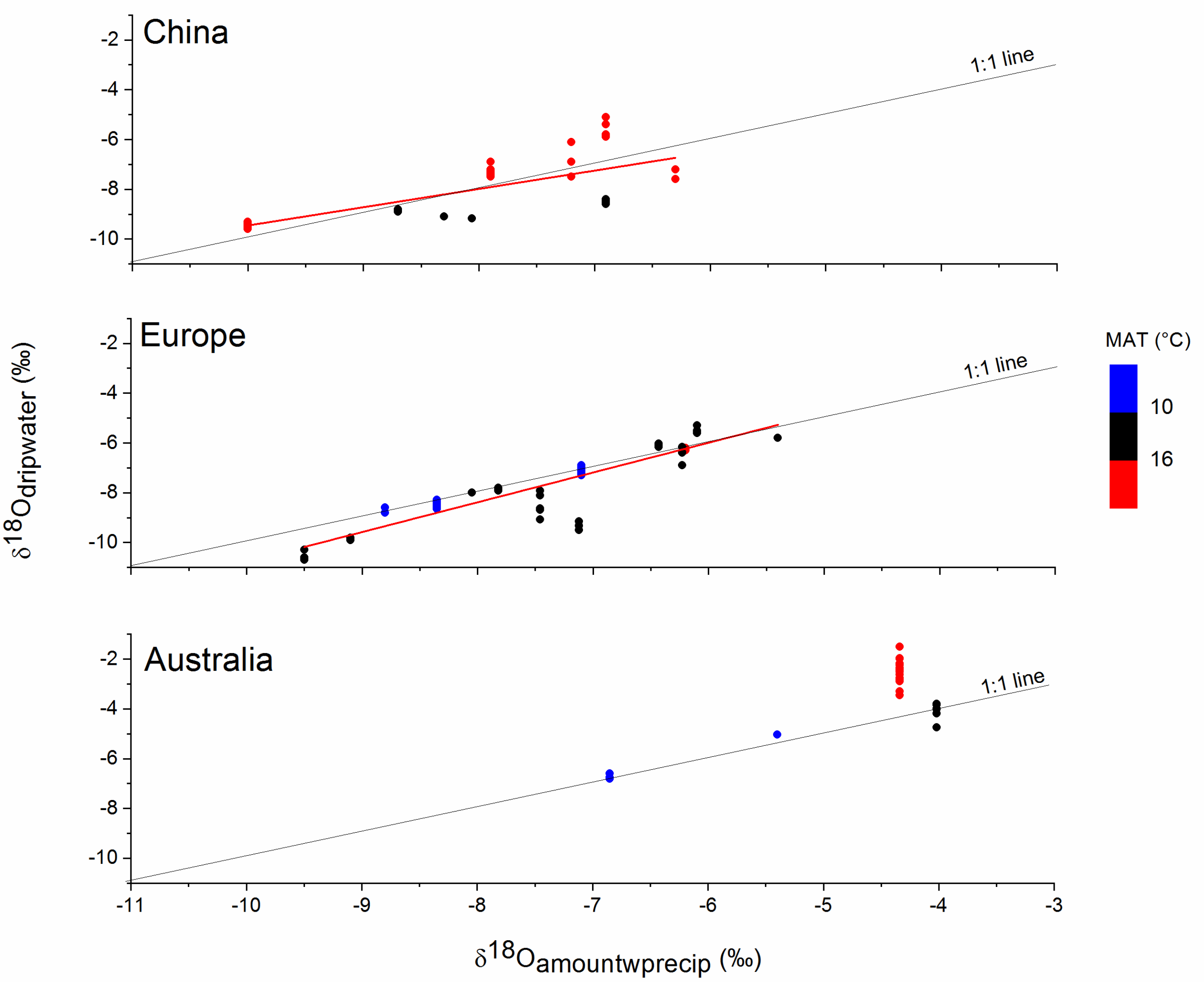

Regional relationships between $\delta^{18}O_{dripwater}$, and $\delta^{18}O_{amountwprecip}$. Regional regression lines are shown in red where they are statistically significant. China: $\delta^{18}O_{dripwater}$ = -2.11 (± 1.05) + 0.74 (± 0.13) $\delta^{18}O_{amountwprecip}$ (‰) r_s = 0.70, $p < 0.05$. Europe: $\delta^{18}O_{dripwater}$ = 1.19 (± 0.59) + 1.20 (± 0.08) $\delta^{18}O_{amountwprecip}$ (‰) r_s = 0.90, $p < 0.01$. Australia: $\delta^{18}O_{dripwater}$ = 2.78 (± 1.01) + 1.38 (± 0.21) $\delta^{18}O_{amountwprecip}$ (‰) r_s = 0.34, p = 0.58. Correlations are Spearman's rank correlation (r_s). Probability values (p), are based on the number of cave sites in the region (Global, n=39; Europe: n= 16; China: n=10; Australia: n=5), rather than number of unique drip waters.

Supplementary Figure 2.

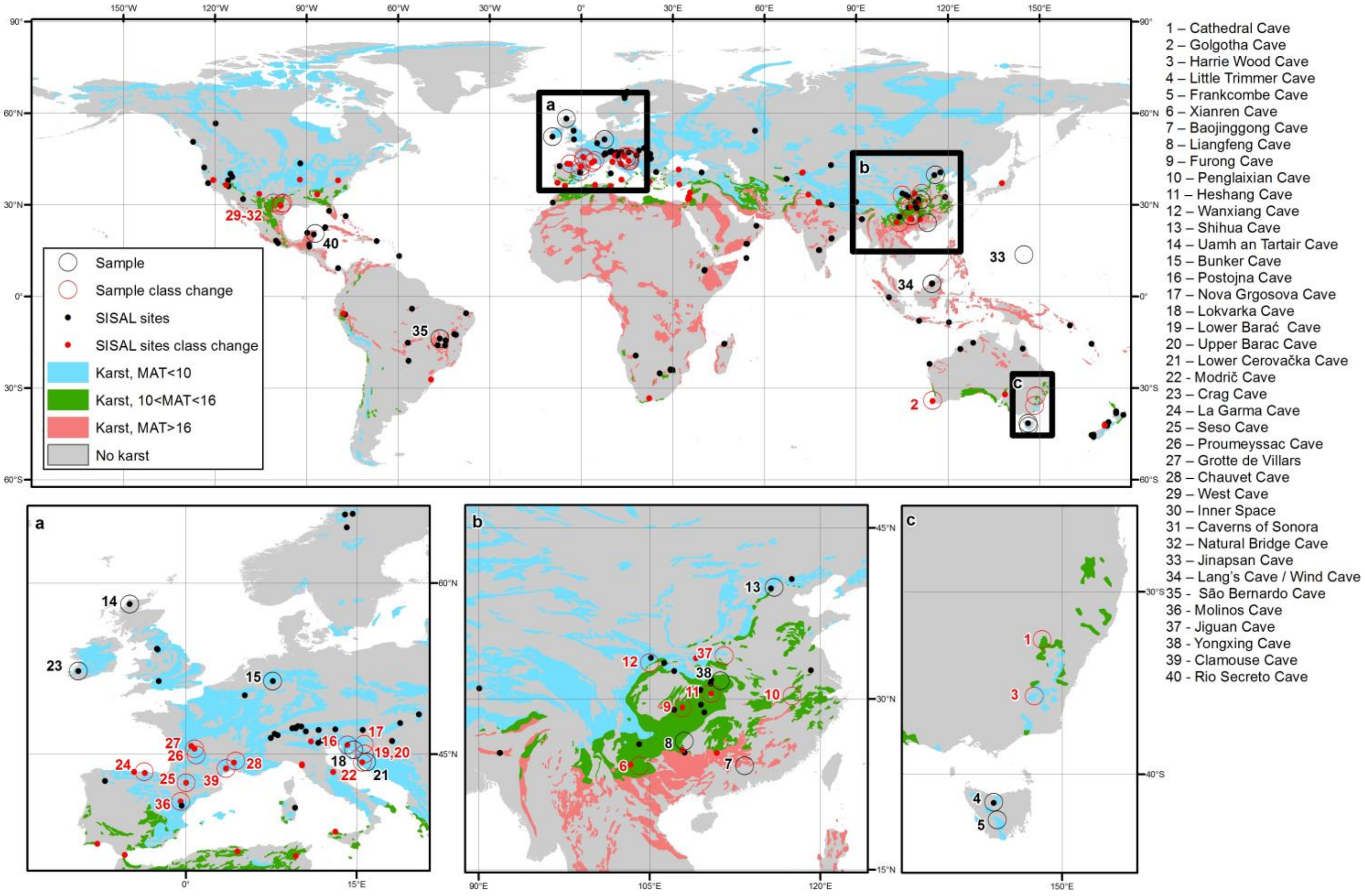

Last Glacial Maximum (LGM) mean annual temperatures (MAT) simulated by the ECHAM5-wiso model. Simulated temperature anomalies have been converted to absolute temperatures adding the modern (2000-2010 CE) climatology from the observational CRU-TS4.01 dataset [22]. Simulated temperature anomalies have been interpolated to the CRU-TS4.01 spatial resolution. Details on the simulation setup can be found in [23,24]. SISAL (Speleothem Isotopes Synthesis and AnaLysis Working Group) sites [25] and samples (Supplemental Data 1) that change between one of the three temperature classes (MAT < 10 °C, 10 < MAT < 16 °C, and MAT > 16 °C) between LGM and modern are shown in red.

Supplementary Figure 3.

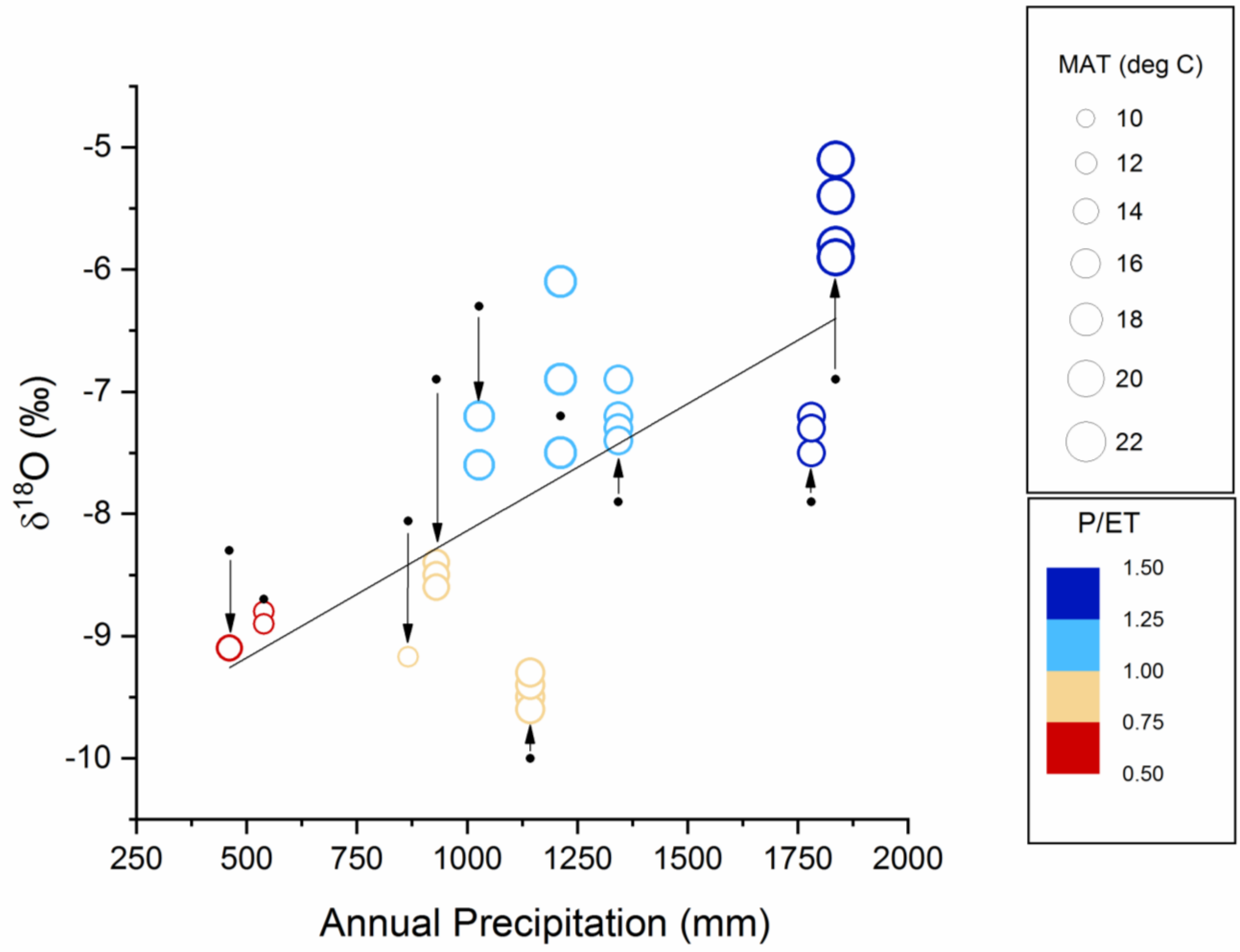

Comparison of annual precipitation and $\delta^{18}O_{dripwater}$ (coloured open proportional circles) and $\delta^{18}O_{amountwprecip}$ (black circles) for the Chinese region. $\delta^{18}O_{dripwater}$ circle diameter is proportional to mean annual temperature, and $\delta^{18}O_{dripwater}$ circle colour represents P/PE. Arrows link $\delta^{18}O_{amountwprecip}$ and $\delta^{18}O_{dripwater}$. In the Chinese region, warmer sites tend to have higher P/ET and $\delta^{18}O_{dripwater} > \delta^{18}O_{amountwprecip}$, and cooler sites tend to have lower P/ET and $\delta^{18}O_{dripwater} < \delta^{18}O_{amountwprecip}$. The relationship between total annual P and $\delta^{18}O_{dripwater}$ is Annual P = 4259.2 (± 307.2) + 269.83 (± 38.71) $\delta^{18}O_{dripwater}$ ($r_s = 0.69$, $p = 0.027$). Probability values (p), is determined using the lowest degrees of freedom (df) based on the number of cave sites in the region (n=10) rather than number of unique drip waters.

Supplementary References

1 Cuthbert, M.O. et al. Drip water isotopes in semi-arid karst: implications for speleothem paleoclimatology. Earth Planet. Sci. Lett. 395, 194-204 (2014).

2 Treble, P.C. et al. Impacts of cave air ventilation and in-cave prior calcite precipitation on Golgotha Cave dripwater chemistry, southwest Australia. *Quatern. Sci. Rev.* **127**, 61-72 (2015).

3 Tadros, C. V. et al. ENSO - cave drip water hydrochemical relationship: A 7-year dataset from south-eastern Australia. *Hydrol. Earth System Sci.* **20**, 4625-4640 (2016).

4 Goede, A., Green, D.C & Harmon, R.S. Isotopic composition of precipitation, cave drips and actively forming speleothems at three Tasmanian cave sites. *Helictite* **20**, 116-126 (1983).

5 Duan, W. et al. The transfer of seasonal isotopic variability between precipitation and drip water at eight caves in the monsoon regions of China. Geochim. Cosmochim. Acta 183, 250-266 (2016).

6 Zhao Jingyao. et al. Variation of δ18O values in the precipitation, cave drip water and modern calcite deposition in Jiguan cave, Henan Province and its atmospheric circulation effect. *Quaternary Sciences (Chinese with English abstract)* **34**, 1106-1116 (2014).

7 Wang, Q. et al. The transfer of oxygen isotopic signals from precipitation to drip water and modern calcite on the seasonal time scale in Yongxing Cave, central China. *Environ. Earth Sci*. **77**, 474 (2018)

8 Fuller, L. et al. Isotope hydrology of dripwaters in a Scottish cave and implications for stalagmite palaeoclimate research. *Hydrol. Earth System Sci.* **12**, 1065-1074 (2008)

9 Richelmann, S. et al. Sensitivity of Bunker Cave to climatic forcings highlighted through multi-annual monitoring of rain-, soil- and dripwaters. *Chem. Geol.* **449**, 194-205 (2017).

10 Dominguez-Villar, D. et al. Ion microprobe $\delta^{18}O$ analyses to calibrate slow growth rate speleothem records with regional $\delta^{18}O$ records of precipitation. *Earth Planet. Sci. Lett.* **482**, 367-376 (2018).

11 Surić, M., Lončarić, R., Bočić, N., Lončar, N. & Buzjak, N. Monitoring of selected caves as a prerequistite for the speleothem-based reconstruction of the Quaternary environment in Croatia. *Quat. Int.* dx.doi.org/10.1016/j.quaint.2017.06.042 (2017).

12 Czuppon, G., Bočić, N., Buzjak, N., Óvári, M. & Molnár, M. Monitoring in the Baracand Lower Cerovacka caves (Croatia) as a basis for the characterisation of the climatological and hydrological processes that control speleothem formation. *Quat. Int.* doi.org/10.1016/j.quaint.2018.02.003 (2018)

13 Baldini, L.M. et al. Regional temperature, atmospheric circulation, and sea ice variability within the Younger Dryas Event cosntrained using a speleothem from northern Iberia. *Earth Planet. Sci. Lett.* **419**, 101-110 (2015).

14 Moreno, A. et al. 2014. Climate controls on rainfall isotopes and their effects on cave drip water and speleothem growth: the case of Molinos cave (Teruel, NW Spain). *Clim. Dyn*. **43**, 221-241 (2014).

15 Genty, D. et al. Rainfall and cave water isotopic relationships in two South-France sites. *Geochim. Cosmochim. Acta* **131**, 323–343 (2014)

16 Pape, J.R., Banner, J.L., Mack, L.E., Musgrove, M. & Guilfoyle, A. Controls on oxygen isotope variability in precipitation and cave drip waters, central Texas, USA. J. Hydrol.385, 203-215 (2010).

17 Partin, J.W. et al. Relationship between modern rainfall variability, cave dripwater and stalagmite geochemistry in Guam, USA. *Geochem., geophy., geosy.* **13**, Q03013 (2012).

18 Partin, J.W., Cobb, K.M., Adkins, J.F., Tuen, A.A. & Clark, B. Trace metal and carbon isotopic variations in dripwater and stalagmite geochemistry from northern Borneo. *Geochem., geophy., geosy.* **14**, 3567-3585 (2013).

19 Moerman, J.W. et al. Diurnal to interannual rainfall $\delta^{18}O$ variations in northern Borneo driven by regional hydrology. Earth Planet. Sci. Lett. 369-370, 108-119 (2013).

20 Moquet, J.S. et al. Calibration of speleothem $\delta^{18}O$ records against hydroclimate instrumental records in Central Brazil. *Global Planet. Change* **139**, 151-164 (2016).

21 Lases-Hernandez, F., Medina-Elizalde, M., Burn, S. and DeCesare, M., 2019. Long-term monitoring of drip water and groundwater stable isotopic variability in the Yucatán Peninsula: Implications for recharge and speleothem rainfall reconstruction. *Geochimica et Cosmochimica Acta*, **246**, 41-59

22 Harris, I., Jones, P. D., Osborn, T. J., & Lister, D. H. Updated high-resolution grids of monthly climatic observations – the CRU TS3.10 Dataset, *International Journal of Climatology*, **34**, 623-642, retrieved from https://crudata.uea.ac.uk/cru/data/hrg/cru_ts_4.01/ on November 2018, 2014.

23 Comas-Bru, L. et al. Evaluating model outputs using integrated global speleothem records of climate change since the last glacial, *Clim. Past Discuss*., https://doi.org/10.5194/cp-2019-25, in review, 2019.

24 Werner, M., Jouzel, J., Masson-Delmotte, V. & Lohmann, G. Reconciling glacial Antarctic water stable isotopes with ice sheet topography and the isotopic paleothermometer, *Nature Communications*, **9**, 3537, https://doi.org/10.1038/s41467-018-05430-y, 2018.

25 Atsawawaranunt, K., Harrison, S., Comas-Bru, L. SISAL (Speleothem Isotopes Synthesis and AnaLysis Working Group) database version 1b. University of Reading. Dataset. http://dx.doi.org/10.17864/1947.189 (2019)
