# Supplementary material for: Global analysis reveals climatic controls on the oxygen isotope composition of cave drip water
Source: Nat Commun. 2019 Jul 5;10:2984. doi: 10.1038/s41467-019-11027-w (PMC6611902; doi:10.1038/s41467-019-11027-w)
Supplement: Supplementary file 3 — Description of Additional Supplementary Files [file 41467_2019_11027_MOESM3_ESM.pdf]

Description of Additional Supplementary Files

File Name: Supplementary Data 1

Description: Mean annual temperature and total annual precipitation are from local measurements reported in the publications, with the exception of total annual rainfall at the Borneo sites, where total annual precipitation data was not reported and gridded data is used. Total annual potential evapotranspiration (PET) and P/PET are sourced from the WorldClim gridded database (see Methods). Recharge-weighted annual average dripwater $\delta^{18}O$ was determined from karst hydrological modelling at European sites. See Supplementary Information for references.
